# 6-Shogaol enhances the anticancer effect of 5-fluorouracil, oxaliplatin, and irinotecan via increase of apoptosis and autophagy in colon cancer cells in hypoxic/aglycemic conditions

**DOI:** 10.1186/s12906-020-02913-8

**Published:** 2020-05-11

**Authors:** Marta Woźniak, Sebastian Makuch, Kinga Winograd, Jerzy Wiśniewski, Piotr Ziółkowski, Siddarth Agrawal

**Affiliations:** 1 Department of Pathology, Wroclaw Medical University, ul. K. Marcinkowskiego, 150-368 Wrocław, Poland; 2 Department of Chemistry, Wroclaw University of Science and Technology, Wrocław, Poland; 3 Department of Biochemistry, Wroclaw Medical University, Wrocław, Poland; 4 Department and Clinic of Internal Medicine, Occupational Diseases, Hypertension and Clinical Oncology, Wroclaw Medical University, Wrocław, Poland

**Keywords:** 5-fluorouracil, 6-shogaol, Autophagy, Chemosensitivity, Colon cancer, Hypoxia

## Abstract

**Background:**

The development and growth of colorectal cancer based on constitutive activation of numerous signaling pathways that stimulate proliferation and metastasis. Plant-derived agents excel by targeting multiple aspects of tumor progression. Previous investigations have shown that ginger derivatives- shogaols possess anti-cancer and anti-inflammatory effects. In the present study, we have examined the anti-cancer effects of 6-shogaol alongside with the most widely used chemotherapeutic agents/regimens in the tumor-like microenvironment conditions.

**Methods:**

Cytotoxicity on two colon cancer cell lines (SW480 and SW620) was measured by MTT test. Apoptosisassay, immunocytochemical and Western blotting analysis for autophagy and apoptosis detection were performed.

**Results:**

Here, we report that 6-shogaol by itself or in combination with chemotherapeutic agents/regimens exerted a cytotoxic effect on CRC cells. Cell death might be linked with the activation of autophagy and apoptosis-related pathways. In the tumor-like microenvironment, which is characterized by hypoxia and glucose starvation, 6-shogaol with chemotherapeutics is significantly more potent than conventional chemotherapy alone.

**Conclusions:**

Collectively, our data suggest that the addition of 6-shogaol to established chemotherapeutic regimens could potentially be a remarkable therapeutic strategy for colorectal cancer.

## Background

Colorectal cancer (CRC) is the second most frequently diagnosed cancer in women and first in men worldwide [1, 2]. Surgery and subsequent chemotherapy are used as the main treatment for three-fourths of patients with colon cancer, but more than 30% of them develop recurrent disease and second malignancy [3, 4]. In the case of metastatic disease, the prognosis is poor with a 5-year survival of less than 10% [5]. Despite the development and approval of biologically targeted agents in the clinic, marginal benefits have been observed in broad patient populations [6]. One of the explanations for this phenomenon can be related to the fact that chemotherapeutics usually block only one component of a pathway and that strategy may not kill the aberrant cancer cell effectively. It is well-established that the development and growth of many cancers, including CRC, are related to constitutive activation of numerous signaling pathways that stimulate proliferation and metastasis, as well as inhibit cell death [7]. Furthermore, the solid tumor microenvironment is characterized by inadequate oxygen and glucose supply [8]. The rapid proliferation of cancer cells results in deficient oxygen levels (less than 2%) and glucose starvation in tumors [9]. Those hallmarks of cancer niche can profoundly affect the cancer cell response in the presence of different chemotherapeutics by increased adaptation to apoptosis and autophagy [10]. Several studies demonstrate that the hypoxic microenvironment was able to effectively induce 5-fluorouracil chemoresistance in colon cancer [11, 12]. Considering that multiple pathways are dysfunctional and during cancer cells growth mutations are accumulated, the most valuable therapeutics should address several targets and present strong effectiveness in sensitizing cancer cells in hypoxic and glucose starvation conditions. Accumulating evidence suggests that plant-derived agents excel by targeting multiple aspects of tumor progression [13, 14].

Ginger (*Zingiber officinale Roscoe*) has been extensively used as a herbal medicine for thousands of years worldwide. It has been applied as an antipyretic, anti-inflammatory and analgesic agent to treat indigestion, infections, digestive tract dysfunctions such as nausea, vomiting, and diarrhea [15]. One significant class of ginger derivatives are shogaols that are found exclusively in dried ginger. Moreover, previous investigations have shown anticancer properties of shogaols; in particular, 6-shogaol can induce cancer cell death through the generation of reactive oxygen species and trigger mitochondrial-dependent apoptosis [16–19]. Therefore, it is indicated that the process of autophagy caused by 6-shogaol is the primary cause of the lung [20], breast [21], and colon [22] cancer cell death.

To address the above-mentioned subjects, we have investigated whether natural plant derivative- 6-shogaol enhances the anticancer effect of the most popular chemotherapeutic agents/regimens used in colon cancer treatment on two human cell lines: SW480 derived from the primary site and SW620 derived from metastatic lymph node site of the same patient. Here, we report the effects of this combined treatment on cancer growth inhibition in the tumor-like microenvironment conditions. The experiments were carried out at hypoxic oxygen concentrations (1%) and in culture medium without glucose. In the present study cell cytotoxicity was verified by MTT viability test. Apoptosis rate was examined using flow cytometry. Moreover, to establish the expression of crucial proteins related to programmed cell death (Bax, Bcl-2, caspase 3) and autophagy (LC-3 I/II, Beclin-1, ATG-7) following cell treatment immunocytochemical staining and Western blotting were performed. In this paper, we discuss the importance of tumor microenvironment conditions in studying the efficacy of combined therapy.

## Methods

### Cell culture and experiment conditions

The human colon cancer cell lines SW620 (kindly provided by prof. Joanna Wietrzyk, Institute of Immunology and Experimental Therapy, the Polish Academy of Sciences, Poland, ATCC no.: CCL-227) and SW480 (Leibniz Institute DSMZ-German Collection of Microorganisms and Cell Cultures, DSMZ, Germany no.: ACC 313) were maintained in RPMI-1640. For the assessment of cytotoxicity effect of 6-shogaol on normal cells, the human normal fibroblast WI38 (kindly provided by Elzbieta Wojdat, Institute of Immunology and Experimental Therapy, the Polish Academy of Sciences, Poland, ATCC no.: CCL-75) were chosen. The cell line was maintained in Eagle’s Minimum Essential Medium. All cell lines were used at early passage (5–12). The base culture media were supplemented with heat-inactivated 10% fetal bovine serum, 1% GlutaMax, and 1% penicillin-streptomycin. Cells were cultured at 37 °C in a 5% CO_2_, and humidified atmosphere to 80% confluence, detached with 0.25% trypsin-EDTA, centrifuged, counted and seeded on plates. Following experiments, cells were maintained in hypoxic and aglycemic conditions. Hypoxia environment was achieved by incubating cells in 1% O_2_, 5% CO_2_ and 37 °C electronically regulated incubator (New Brunswick Galaxy 48R, Eppendorf, Hamburg, Germany) and glucose starvation by the culture of cells in complete growth medium without glucose (RPMI 1640, no glucose, cat. no. 11879020). After 24 h of fresh medium with drugs, and 6-shogaol was added to the wells, then cells were treated for a further 48 h. Cell culture reagents were purchased from Gibco, (Thermo Fisher Scientific Inc., Waltham, Massachusetts, USA).

### 6-shogaol and drug preparation

6-shogaol was purchased from MedChemExpress (Sollentuna, Sweden), 5-fluorouracil (FU) was purchased in Sigma-Aldrich (Merck KGaA, Darmstadt, Germany), oxaliplatin (OXA), irinotecan (IRI) in Selleckchem (Munich, Germany). Stocks of 6-shogaol and chemotherapeutics were prepared according to the manufacturer’s instructions, divided into aliquots and stored at − 80 °C. For further experiments, reagents were freshly diluted to the desired concentrations in the complete culture medium. Controls were performed for all experiments using cells grown in standard culture medium with a maximum of 0.05% DMSO (BioShop Canada Inc., Ontario, Canada) if reagents were solubilized in DMSO.

### MTT assay

The cytotoxicity of 6-shogaol and chemotherapeutics in SW620, SW480, WI38 cells was measured by the 3-(4,5-dimethylthiazol-2-yl)-2,5-diphenyltetrazolium bromide (MTT) reduction assay (Sigma-Aldrich). Cells were seeded at a density of 1 × 10^4^/well in 96-well culture plates and treated as described in experimental conditions with 6-shogaol and different chemotherapeutics combinations for 48 h. Different doses of 6-shogaol were experimentally determined for further studies on SW480, SW620, and WI38. Tested concentrations were 5, 10, 20, 40, 80 μM. The dose of chemotherapeutics was established based on the previous studies and tested on cancer cells: FU 125 μM, IRI 75 μM, OXA 50 μM. The same concentrations of drugs were used when the combination of chemotherapeutic (FU + IRI), (FU + OXA), (FU + IRI + OXA) was applied. After treatment time, cells were washed, MTT solution was added to the wells to a final concentration of 0.5 mg/ml and incubated at 37 °C for 4 h. The violet formazan crystals were solubilized with 100 μl DMSO (Sigma-Aldrich) for 30 min. The optical absorbance (A) was measured at 490 nm using a BioTek ELX800 multi-well reader (BioTek, Winooski, VT, USA). The absorbance of the control group was determined as 100% cell viability. The percentage of viable cells (VC) was calculated according to the formula: VC (%) = (A of experimental group/A of the control group) × 100%. All MTT assays were repeated three times, and figure graphs represent mean from three experiments. For further experiments, SW480 cell line was chosen and 6-shogaol in the concentration of 25 μM and FU in dose 125 μM were used to evaluate migration ability, apoptosis and autophagy response to the combined/alone treatment.

### Apoptosis detection by flow cytometry

The ratio of live, early apoptotic, late apoptotic and dead cells was measured using Annexin V Apoptosis Detection Kit APC (Thermo Fisher Scientific Inc.). Cells were seeded at a density of 1 × 10^6^/well in 6-well culture plates and treated as described in experimental conditions with 6-shogaol with/without FU for 48 h. The cells were washed with PBS, detached from each well of 6-well plate by Gibco™ Trypsin-EDTA reagent and again washed with PBS. After centrifugation cell pellets from each tube were resuspended in 1 ml of 1XBinding Buffer. Then the amount of 100 μL of each cell sample was added to a new 1,5 ml tube and mixed with 5 μL of APC fluorochrome-conjugated Annexin V and 5 μL of Propidium Iodide Staining Solution. After 20 min of incubation at room temperature in the dark, samples were gently mixed and then loaded onto BD Accuri 6 Analyzer (Becton, Dickinson and Company, NJ, USA). Each sample was tested in triplicates. The results were analyzed using BD Accuri 6 Plus Software (Becton, Dickinson and Company).

### Immunocytochemistry analysis

An apoptotic and autophagy protein expression after 6-shogaol with/without FU therapy was evaluated by immunocytochemistry. For experiments, SW480 cells were seeded on 8 Chamber Eppendorf Cell Imaging Slides (Eppendorf). Immunocytochemistry was performed using reagents from Agilent (Santa Clara, CA, USA). Next day 6-shogaol in dose 25 μM, FU 125 μM and 6-shogaol+FU were added to the chambers and incubated for 48 h. Following incubation, cells were washed twice in PBS and fixed in 4% paraformaldehyde (Polysciences, Warrington, PA, USA) for 10 min. Chambers with cells were washed in PBS, permeabilized in 0,1% Tween 20 for 10 min, washed in PBS, then endogenous peroxidases were blocked. Following incubation with protein blocking buffer, primary antibodies were applied for overnight incubation at 4 °C. Autophagy antibodies used in the study: rabbit polyclonal anti-LC3 diluted 1:150 (Sigma-Aldrich); rabbit polyclonal anti-Atg7 diluted 1:200 (Santa Cruz Biotechnology, Inc., Santa Cruz, CA, USA); mouse polyclonal anti-Beclin-1 diluted 1:200 (Sigma-Aldrich). Apoptotic antibodies used in the study: anti-Bcl-2 (Sigma-Aldrich) diluted 1:200; anti-Bax (Sigma-Aldrich) diluted 1:200 and anti-Caspase-3 (Sigma-Aldrich) diluted 1:200.

The next day, the slides were washed with PBS and incubated for 1 h with a secondary antibody, dilution 1:100). Then the slides were rinsed twice with PBS and stained with 3,3′-diaminobenzidine (DAB) in chromogen solution. Finally, cells were counterstained with Mayer’s hematoxylin, washed and mounted in Faramount Aqueous Mounting Medium (Sigma-Aldrich). The negative controls were obtained by omitting the first antibodies. Photographs were taken by a light microscope fitted with a digital camera (Nikon) at magnifications of 400x.

The protein expression was defined as the number of the intensity and quantity of DAB-stained cells in the immunoreactivity score (IRS):

- 0 = negative or weak staining of ≤10% cells
- 1 = weak staining of 11 to 30% cells
- 2 = moderate staining of 31 to 70% cells
- 3 = strong staining of ≥71% cells

### Western blotting analysis

For autophagy and apoptotic protein detection, the Western blotting analysis was performed on the SW480 cancer cell line. SW480 cells were seeded at a density of 3,5 × 10^5^/well in 6-well culture plates and treated in combination or alone with 6-shogaol and FU for 48 h. For cell lysates, preparation suspension cells in growth medium were collected, washed and mixed with adherent were washed twice with cooled PBS. In the next step, cell lysates were prepared using RIPA buffer containing protease and phosphatase inhibitors (1% cocktails, Sigma-Aldrich,). The lysates were incubated with low agitation for 30 min at 4 °C and then centrifuged at 16000 g for 30 min. The protein level was measured at 280 nm using the Qubit Protein Assay for the Qubit Fluorimeter (Invitrogen, Thermo Fisher Scientific Inc.). Protein extracts-equivalent of 25 μg were separated by 4–12% SDS-polyacrylamide gel electrophoresis (Invitrogen, Thermo Fisher Scientific Inc.) After electrophoresis, proteins from the gel were electrotransferred on a 0.2 μm nitrocellulose (Bio-Rad Laboratories) using Trans-Blot® Turbo™ Transfer System (30 min, Standard Run). The membrane was blocked for 1 h at room temperature with 5% w/v Nonfat Dry Milk bovine (Cell Signaling Technology, Danvers, MA, USA) diluted in TBST (50 mM Tris-HCl, 150 mM NaCl, 0.1% Tween-20, pH 7.5). Subsequently, after four 5 min cycles of washing in TBST buffer, the membrane was incubated for additional 2 h at RT in antibodies for autophagy: rabbit polyclonal anti-LC3 diluted 1:350 (Sigma-Aldrich); rabbit polyclonal anti-Atg7 diluted 1:500 (Santa Cruz Biotechnology, Inc.); mouse polyclonal anti-Beclin-1 diluted 1:250 (Sigma-Aldrich) and antibodies for apoptosis: anti-Bcl-2 (Sigma-Aldrich) diluted 1:400; anti-Bax (Sigma-Aldrich) diluted 1:450 and anti-Caspase-3 (Sigma-Aldrich) diluted 1:400. For normalization of the loading differences, a monoclonal antibody against β-actin was used as a control. The membranes were washed three times with TBST and incubated with a horseradish peroxidase-labeled secondary goat anti-rabbit antibody (Santa Cruz Biotechnology, Inc.) or anti-mouse IgG, HRP-linked Antibody for 1 h at room temperature and after that washed three times again with TBST. The chemiluminescent detection of proteins was performed using HRP conjugates (horseradish peroxidase) and AP conjugates (alkaline phosphatase) (Bio-Rad). The image of the specific protein bands was documented by Azure C600 (Bio-Rad).

### Statistical analysis

Unless otherwise stated, data are presented as mean ± SD. Where appropriate, the results were analyzed using ANOVA followed by Fischer’s protected least significant differences or Scheffe’s test. A *p*-value of < 0.05 was considered statistically significant.

## Results

### 6-shogaol is selective for cancer cells over normal healthy cells

**Fig. 1:**
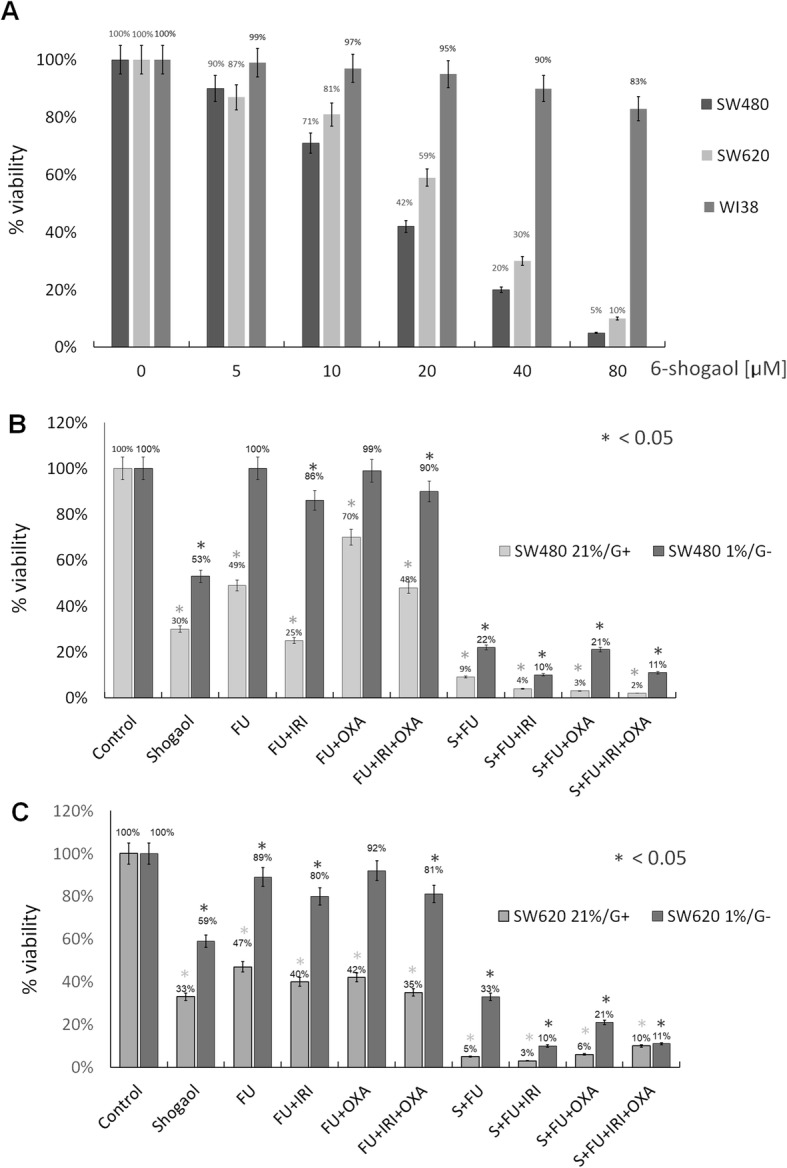
Results of cell viability measured by MTT assay of cell lines. **a** Dose-depended bars for SW480, SW620, WI38 cells after 48 h incubation with 6-shogaol. **b** SW480 cell line viability in normoxia conditions (21% of oxygen, 5% CO_2_, medium with glucose) and hypoxic conditions (1% of oxygen, 5% CO_2_, glucose-free medium) after 48 h of incubation with 6-shogaol (25 μM), different chemotherapeutics (FU 125 μM, IRI 75 μM, OXA 50 μM) alone and in combination with 6-shogaol. **c** SW620 cell line viability in normoxia conditions (21% of oxygen, 5% CO_2,_ medium with glucose) and hypoxic conditions (1% of oxygen, 5% CO_2_, glucose-free medium) after 48 h of incubation with 6-shogaol (25 μM), different chemotherapeutics alone (FU 125 μM, IRI 75 μM, OXA 50 μM) and in combination with 6-shogaol. Control-cells incubated in a complete culture medium, Shogaol- 6-shogaol, FU-fluorouracil, IRI-irinotecan, OXA-oxaliplatin, medium with glucose (G+), glucose-free medium (G-). Bars show mean from three experiments. * *p* < 0.05

A dose-response study was performed with 6-shogaol in SW480 and SW620 adenocarcinoma cells and matched fibroblasts WI38. We found that 6-shogaol caused a significantly higher reduction of viability in both cancer cell lines as compared to the normal cells. It inhibited the growth of cancer cells in a dose-dependent manner, exhibiting cytotoxicity by 95% in SW480 and 90% in SW620 at 80 μM concentration, whereas the viability of WI38 was decreased only by 17% (Fig. 1a). For SW480 and SW620 cell line, a decrease by 42 and 59% of viable cells were observed at a dose of 20 μM which was chosen in all subsequent studies (Fig. 1a).

### 6-shogaol in combination with chemotherapeutics increases cell cytotoxicity

The next set of MMT analysis was to examine whether 6-shogaol given together with different chemotherapeutics would affect cancer cell growth. The results showed that although 6-shogaol, FU, FOLFIRI (FU + irinotecan), FOLFOX (FU + oxaliplatin), FOLFOXIRI (FU + oxaliplatin+irinotecan) were all potent in significantly decreasing of SW480 or SW620 cell viability ranging from 30 to 65%, treatment with 6-shogaol together with chemotherapeutics caused increased cytotoxicity (98%), when compared with the control samples (statistical significance between controls was *p* < 0.05). Taken together, the results suggest that the treatment of 6-shogaol with chemotherapeutic agents increases the cytotoxic effect in CRC cells. Also, the effect is not cell-specific since it was observed in both SW480 primary site and SW620 metastatic cancer cells.

### 6-shogaol inhibits cell growth in the tumor microenvironment

The tumor microenvironment is mainly characterized by hypoxia (1% oxygen level) and glucose starvation, whereas regular culture represents conditions of high oxygen tension (21%) and glucose levels (4.5 g/L). It was observed that hypoxia displayed a greater risk of tumor advancement and metastasis [23]. Furthermore, the tumor microenvironment might reduce the effectiveness of chemotherapy [24]. In this study, we simulated the tumor microenvironment and evaluated its impact on the activity of 6-shogaol and chemotherapeutics. Figure 1b and c present SW480 and SW620 cells, respectively, in standard culture conditions and the mimicked tumor microenvironment. In both cell lines, we observed that 6-shogaol inhibits cell viability, whereas chemotherapeutics showed no or very weak cytotoxicity in the tumor-like microenvironment. The cytotoxicity of 6-shogaol in both cell lines in hypoxia and aglycemic conditions together with chemotherapeutic was dramatically increased and statistically significant (*p* < 0.05) in each combination (FU, FU-IRI, FU-OXA, FU-IRI-OXA) in comparison to controls.

### 6-shogaol with fluorouracil enhances apoptosis and cell death

**Fig. 2:**
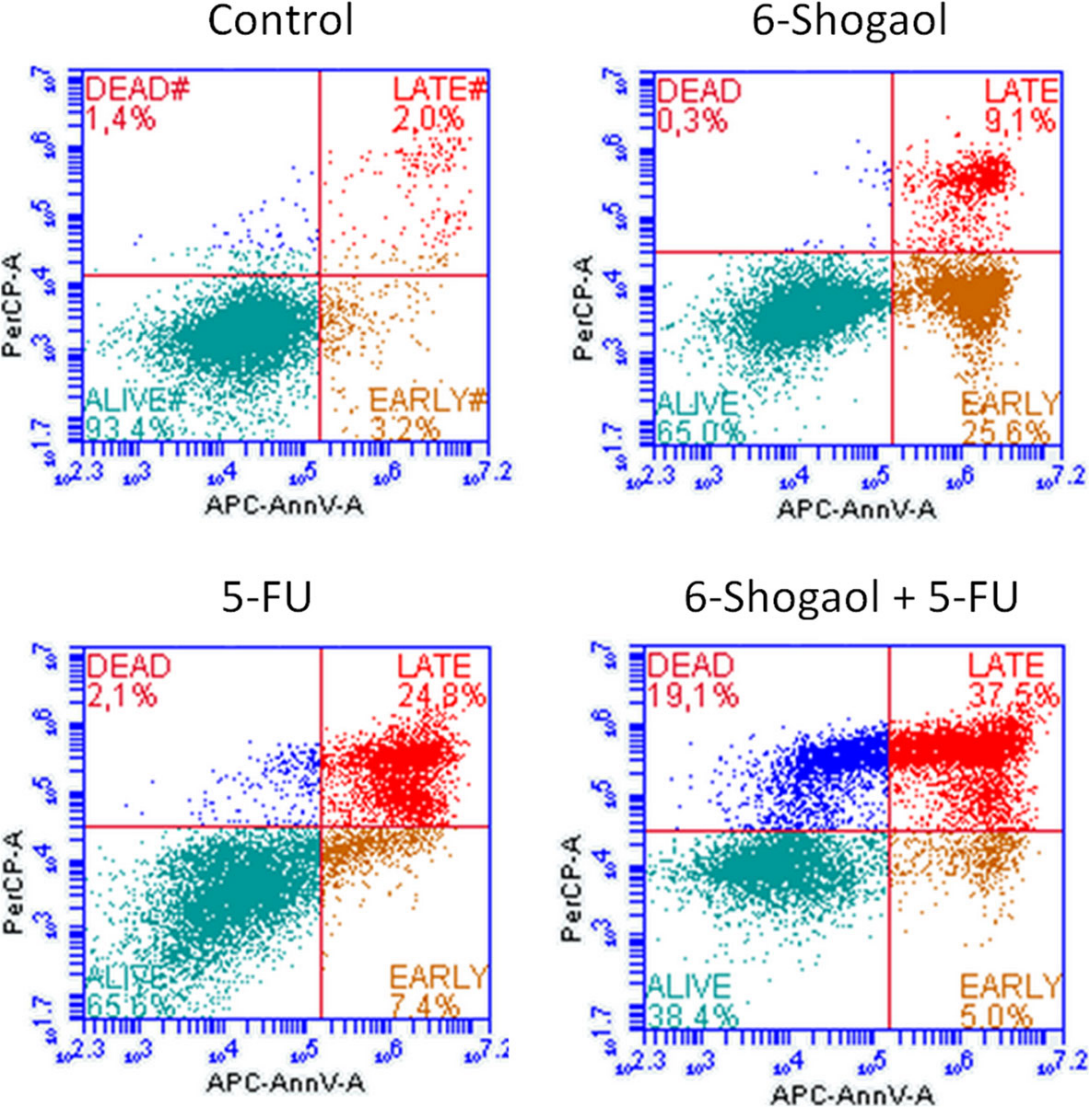
Dot plots presenting alive, early, late apoptotic and dead SW480 cells measured by flow cytometry after treatment with 6-shogaol, FU alone and in combination with both. Cells were stained after 48 h of incubation using Annexin V-APC conjugated fluorochrome and propidium iodide

For the evaluation of the cell death caused by 6-shogaol and FU in SW480 cells, flow cytometry analysis was applied (Fig. 2). The identification of early, late apoptotic and necrotic cells, reveals that either 6-shogaol and fluorouracil alone caused a similar effect of programmed cell death (around 35% of cells underwent apoptosis). Interestingly, the combination of 6-shogaol and FU slightly increased apoptosis over 6%, but the enhancement of cell death is also connected to the increase of necrosis ratio (nearly 20%). Therefore over 65% of the SW480 cells underwent cell death.

### 6-shogaol induces cell apoptosis and autophagy response

**Fig. 3:**
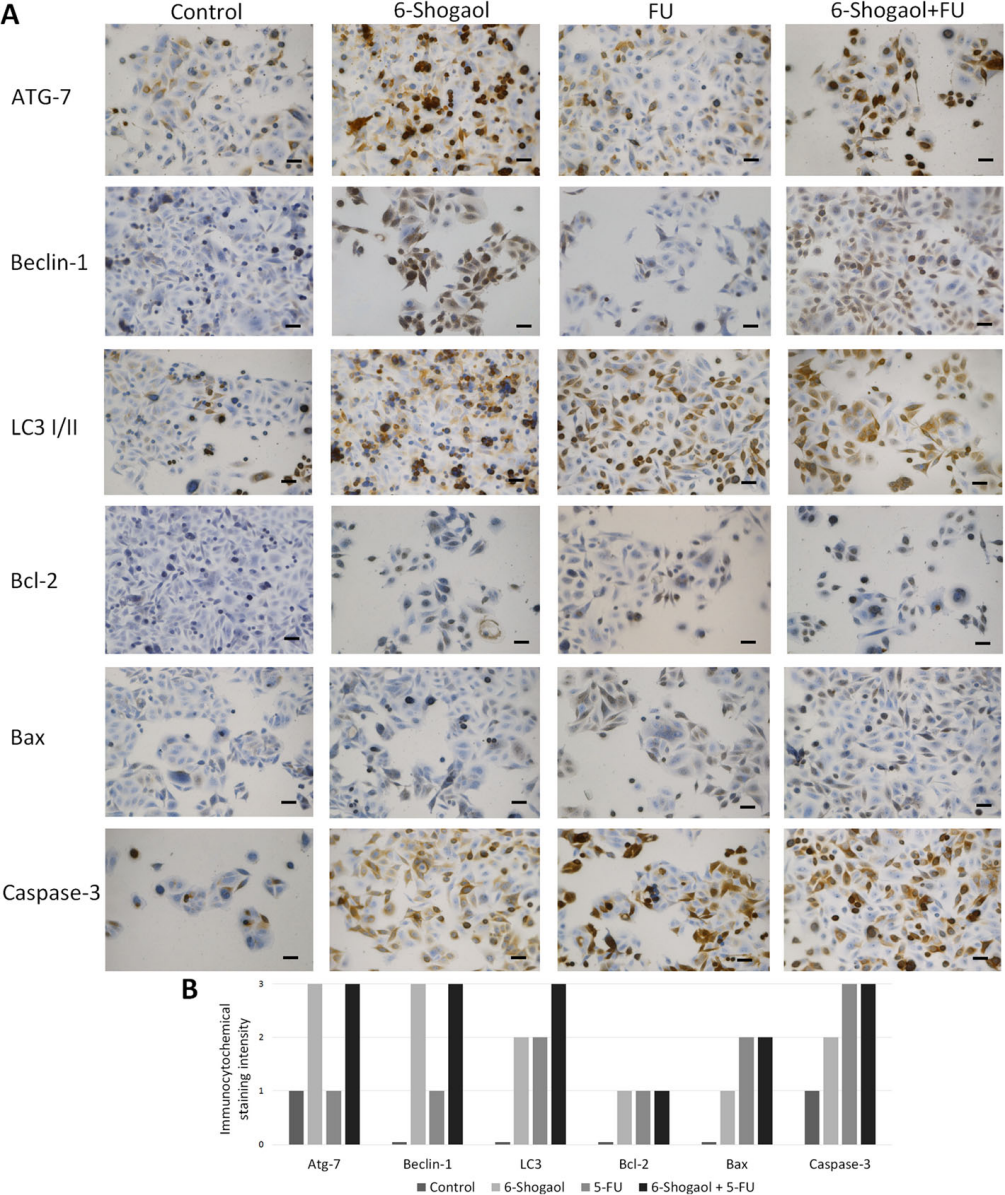
A. Representative images of the immunocytochemical analysis of the chosen autophagy and apoptosis-related proteins in SW480 cell line in four conditions: control, incubation with 6-shogaol only, incubation with 5-FU only and treatment with 6-shogaol + 5-FU together. B. Results of immunocytochemical analysis of SW480 cell line and the chosen autophagy and apoptosis-related protein expression calculated by immunoreactivity score. Abbreviations: 0- no staining, 1- weak staining, 2-moderate staining, 3-strong staining. Control- cells cultured in complete culture medium for 48 h, 6-Shogaol- cells incubated with the compound in dose 25 μM, FU- cells treated with the drug in concentration 125 μM, 6-Shogaol + FU- cells treated with the compound and the drug together. Scale bar = 50 μm.

Since apoptosis and autophagy induction is the principal cancer therapy targets, we have examined if the current proposed treatment of 6-shogaol with a chemotherapeutic also leads to CRC cell death via the aforementioned pathways. To assess the crosstalk between autophagy and apoptosis in 6-shogaol and FU-treated colon cancer cells, three autophagy (LCI/II, Beclin-1, Atg-7) and three apoptosis-related proteins (Bcl-2, Bax, Caspase-3) were selected for the immunocytochemical evaluation (Fig. 3).

**Fig. 4:**
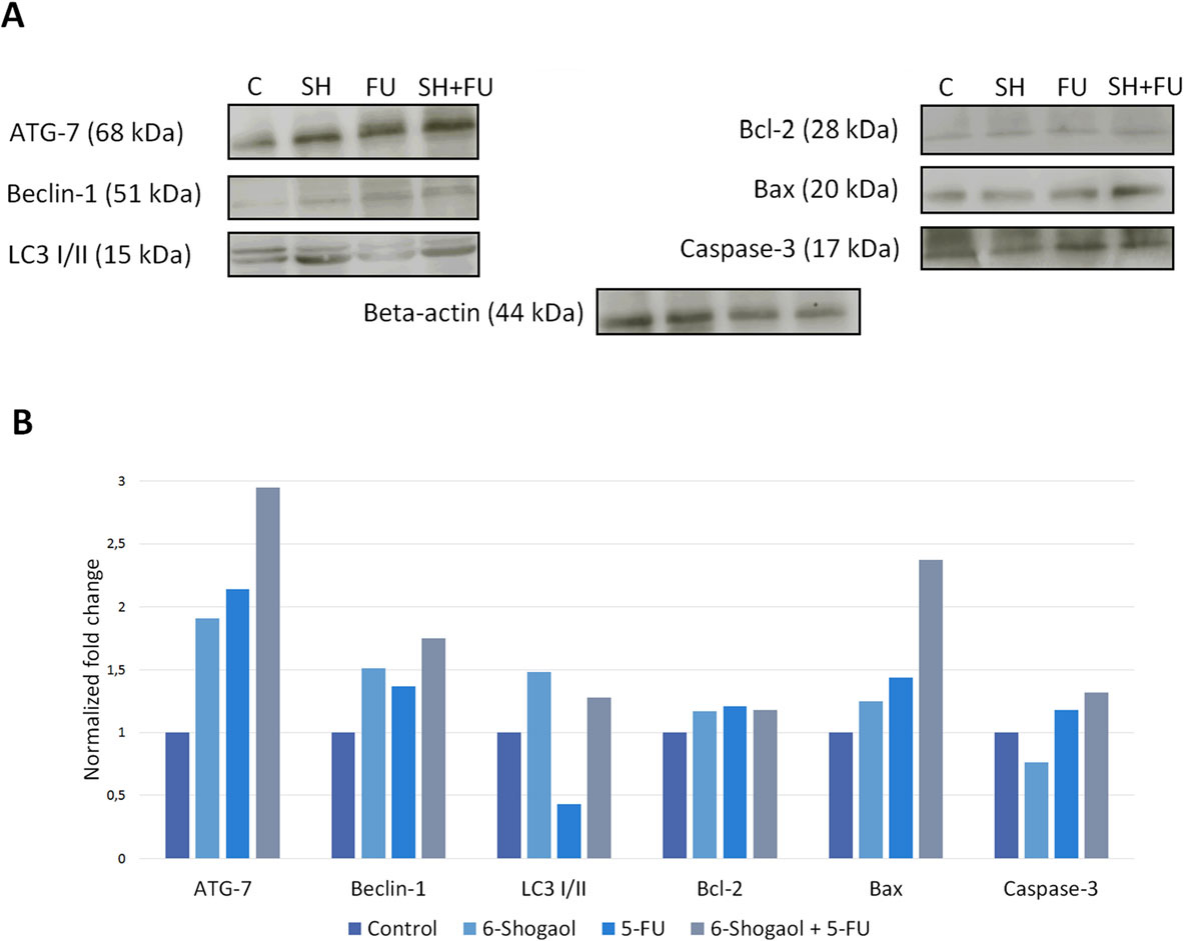
Detection of the protein bands from SW480 cell lysates evaluated by Western blotting analysis. The beta-actin band represents internal control of the protein level in the samples. Quantification of Western blotting analysis for Atg-7, Beclin-1, LC3, Caspase-3, Bcl-2 and Bax protein level in control, 5-fluorouracil and/or 6-shogaol treated SW480 using ImageJ software

The key finding was that following 6-shogaol treatment the autophagy markers were strongly expressed in treated cells (IRS = 3 for Atg-7, Beclin-1, IRS = 2 for LC3) in comparison with control cells where there was no staining observed (IRS = 0). Low immunoreactivity score for FU-only treated cells was observed (IRS = 1 for Atg-7 and Beclin-1; IRS = 2 for LC3). All autophagy proteins showed a strong expression pattern in 6-shogaol+FU treated cells. We found that the strongest immunoreactivity staining was observed in FU-alone and in FU + 6-shogaol treated cells (IRS = 2 for Bcl-2 and IRS = 3 for caspase-3 and Bax). In sharp contrast, in control and 6-shogaol-treated cells, weak staining was noted. To confirm immunocytochemical validation, Western blotting detection of the proteins involved in apoptotic and autophagy response was performed. Western blot from SW480 protein lysates shows the same pattern of protein band occurrence as it was observed in immunocytochemical studies (Fig. 4).

**Fig. 5:**
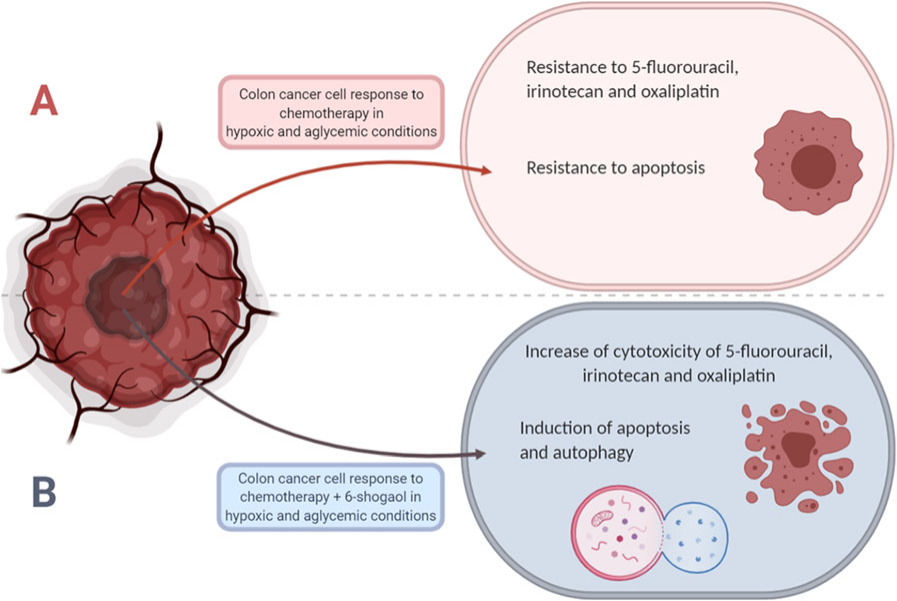
Natural plant derivative 6-Shogaol as a promising agent for enhancing cytotoxicity and overcoming drug resistance to chemotherapy in hypoxic and aglycemic conditions

## Discussion

CRC is one of the most prevalent malignancies and one of the principal world’s leading reason of cancer-related deaths [23]. In patients with advanced cancer, the treatment result is poor; studies indicate that only one out of two patients will respond to the traditional chemotherapy [24, 25]. Therefore, it is highly desirable to validate nontoxic agents that could improve upon the standard chemotherapeutic regimen. Presently, nearly half of the FDA approved therapies are obtained from plants [26]. Many of these herbal substances have antioxidant, anti-inflammatory and anticarcinogenic qualities that are valuable in CRC treatment [27]. An escalating number of studies have revealed that 6-shogaol, an active constituent of dried ginger, has potential anti-tumor activity in multiple cancers. However, up to date, reliable data on the efficacy of 6-shogaol in combination with traditional chemotherapy drugs in the tumor microenvironment is unavailable.

Our study shows that 6-shogaol is highly active against CRC cells derived either from the primary site and the metastatic site cells. 6-shogaol exhibited a higher cytotoxic effect on SW480 in contradistinction to SW620, which may suggest this natural plant derivate is more potent against primary tumors. MTT analysis indicated that 6-shogaol affects cancer cell viability with an IC_50_ value close to 20 μM. The IC_50_ values of the present study for 6-shogaol are in accordance with the available published research [16, 28]. Notably, 6-shogaol preferentially annihilates cancer cells while showing no toxicity in noncancerous cells in vitro in the dose as mentioned earlier. A similar observation was reported in previous studies [29]. Moreover, we found that that 6-shogaol alone and in combination with fluorouracil significantly attenuates the proliferation ability in colon cancer cells within 24 h, which is of particular significance in large and metastatic cancers.

Our current data, for the first time, reveal that 6-shogaol together with chemotherapeutic agents and regimens (FOLFOX, FOLFIRI, FOLFOXIRI) is more potent in inhibiting the growth of SW480 and SW620 colon cancer cells. In all combinations of the chemotherapeutic agents (FU, oxaliplatin, irinotecan), the addition of 6-shogaol increased cytotoxicity of the anti-cancer agents in tumors microenvironment conditions. FU is a pyrimidine analog modified intracellularly into several metabolites, that incorporates into DNA and RNA strands, eventually resulting in cell cycle arrest and apoptosis [30]. Oxaliplatin exerts a suppressive effect by forming DNA inter- and intrastrand cross-links to inhibit replication and transcription, following in apoptosis [31]. Irinotecan, originally isolated from the Tibetan plant Camptothecaacuminata, generates S-phase-specific cell killing by inhibiting topoisomerase I and resulting in apoptosis [32]. The primary mechanism of 6-shogaol inhibiting cancer cell growth and spread is also contributing to apoptosis [15]. The immunocytochemistry and Western blotting analysis revealed that caused a marked activation of apoptosis-related proteins. Caspase and Bcl-2 families play an essential role in the process of programmed cell death. Notably, the activation of caspase 3, a primary protein in the induction of apoptosis, is regulated by the Bcl-2 protein family [33]. The activation of the intrinsic pathway depends on the levels of the apoptotic inhibitor protein Bcl-2 and the proapoptotic protein Bax. Earlier studies have shown that 6-shogaol affects the balance of Bcl-2 family members and promotes the expression of apoptotic activators: Bax and procaspases 3, 8, and 9 [16]. In our study, a combination of 6-shogaol and FU resulted in a high expression of proapoptotic proteins. However, the expression of caspase 3 and Bax was found to be lower in 6-shogaol-only treated cells.

Autophagy is a lysosomal degradation process characterized by the production of autophagosomes. During the process, cytoplasmic content and organelles are separated by an isolation membrane. Merging of this double-membraned vesicle forms an autophagosome, which eventually blends with a lysosome. Autophagosome formation is dependent on the activity of Atg proteins, of these, the Beclin 1 complex, the Atg7 and light chain 3 (LC3) systems act successively during the nucleation and enlargement of the autophagosomal membrane. Cancer cells can undergo programmed cell death through autophagy, which, unlike apoptosis, is characterized by the accumulation of autophagic vacuoles [34]. Radiation and several chemotherapeutic agents induce autophagy [35]. The major finding was that following 6-shogaol treatment; the autophagy markers were strongly overexpressed in CRC cells. The results indicate that 6-shogaol may enhance the chemotherapeutic agents used in CRC therapy through autophagic cell death.

Accumulating evidence points out that cells that reside in large, solid neoplasms are under stress conditions caused by a lack of angiogenesis [36]. It is implied that the tumor microenvironment could influence the potency of anticancer therapeutics. In this sense, we further studied the inhibitory action of 6-shogaol in hypoxia and glucose starvation. The key finding was that in the tumor-like microenvironment 6-shogaol exhibited strong anticancer activity, whereas 5FU, oxaliplatin, and irinotecan showed no or very weak cytotoxicity alone. Hypoxia is usually observed to coexist with low glucose in tumors [37]. Thus, cancer cells cultured in hypoxic and aglycemic conditions may respond differently and exhibit resistance to chemotherapeutic agents used in this study. Taken together, 6-shogaol might be potentially applied to overcome drug resistance to chemotherapy occurring in the tumor microenvironment (Fig. 5). Further in vitro and in vivo analysis are mandatory to evaluate the underlying mechanism of this phenomenon.

## Conclusion

In conclusion, our study reveals that 6-shogaol by itself or in combination with chemotherapeutic agents/regimens exerted a cytotoxic effect on CRC cells. Cell death might be linked with the activation of autophagy and apoptosis-related pathways. We highlighted the importance of the tumor microenvironment on drug efficacy and found that 6-shogaol is significantly more potent in cancers ‘native’ environment than conventional chemotherapy. Collectively, our results suggest that the addition of 6-shogaol to established chemotherapeutic regimens could potentially be a remarkable therapeutic strategy in colorectal cancer therapy.

## Supplementary information

**Additional file 1.**

**Additional file 2.**

**Additional file 3.**

**Additional file 4.**

## Data Availability

All data generated or analyzed during this study are included in this published article.

## Abbreviations

CRC: colorectal cancer
FU: 5-fluorouracil
OXA: oxaliplatin
IRI: irinotecan
DMSO: dimethyl sulfoxide
VC: viable cells
TBST: tris buffered saline with Tween-20
HRP: horseradish peroxidase
AP: alkaline phosphatase
FDA: Food and Drug Administration
