# Supplementary figures and images for: 6-Shogaol enhances the anticancer effect of 5-fluorouracil, oxaliplatin, and irinotecan via increase of apoptosis and autophagy in colon cancer cells in hypoxic/aglycemic conditions
Source: BMC Complement Med Ther. 2020 May 11;20:141. doi: 10.1186/s12906-020-02913-8 (PMC7216385; doi:10.1186/s12906-020-02913-8)

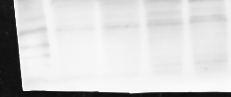

Supplement: Supplementary file 1 — Additional file 1. [file 12906_2020_2913_MOESM1_ESM.tif]

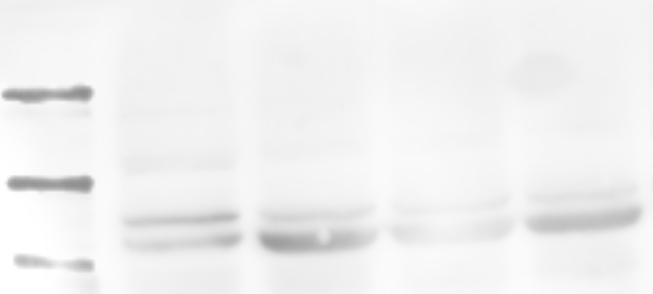

Supplement: Supplementary file 2 — Additional file 2. [file 12906_2020_2913_MOESM2_ESM.tif]

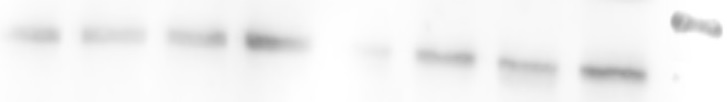

Supplement: Supplementary file 3 — Additional file 3. [file 12906_2020_2913_MOESM3_ESM.tif]

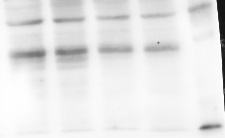

Supplement: Supplementary file 4 — Additional file 4. [file 12906_2020_2913_MOESM4_ESM.tif]
